# Implicit Neural Representation with Dead-Free Linear Unit for Remote Sensing Images

**DOI:** 10.3390/s26082370

**Published:** 2026-04-12

**Authors:** Yi Lu, Chang Lu, Dongshen Han, Donggeon Kim, Mingming Zhang, Rizwan Qureshi, Caiyan Qin

**Affiliations:** 1 School of Mathematical Sciences, Capital Normal University, Beijing 100088, China; 2230501004@cnu.edu.cn; 2 School of Computing, Kyung Hee University, Yongin 17104, Republic of Korea; luchang@khu.ac.kr (C.L.); han-0129@khu.ac.kr (D.H.); dgk1012dgk@khu.ac.kr (D.K.); 3 School of Robotics and Advanced Manufacture, Harbin Institute of Technology, Shenzhen 518055, China; mmzhang@hit.edu.cn; 4 MoSA Labs, Department of Computer Science, Salim Habib University, Karachi 74900, Pakistan; engr.rizwanqureshi786@gmail.com

**Keywords:** Implicit Neural Representation, remote sensing, rectified linear unit

## Abstract

As a crucial component of multimodal sensing in modern AI agents, remote sensing images have attracted significant attention, for which neural representation is a promising direction. Implicit Neural Representations (INRs) using Multi-Layer Perceptrons (MLPs) have the ability to model images by learning an implicit mapping from pixel coordinates to pixel intensities. This paper revisits the ReLU activation function, a widely adopted non-linearity known for its dead region on the negative axis, within the context of MLP-based INRs. We introduce the Dead-Free Linear Unit (DeLU), a novel activation function that leverages a linearly transformed absolute value to eliminate inactive regions. By combining dead-free non-linearity with adaptive linear scaling, DeLU enhances the expressiveness of INR architectures, particularly those employing periodic activations. Extensive experiments across multiple remote sensing datasets, including LandCover.ai, LoveDA, INRIA, UAVid, and ISPRS Potsdam, validate the efficacy of our proposed method.

## 1. Introduction

Modern AI agents are empowered with multimodal sensing, for which remote sensing images play an important role and have attracted significant attention for Implicit Neural Representations (INRs) [1,2]. INRs have emerged as a robust framework for establishing continuous mappings between spatial coordinates and their associated signal attributes, enabling the parameterization of diverse data modalities, including visual imagery, acoustic waveforms, and volumetric scenes [3,4,5]. This paradigm shift in data representation has catalyzed significant interdisciplinary research, particularly in applications such as image compression, novel view synthesis, and geometric reconstruction [6,7,8,9,10]. At its core, an INR implements a continuous functional mapping through multilayer perceptrons (MLPs) [11,12]. Recent advancements in INR optimization have focused on enhancing the spectral fidelity of learned representations. A seminal approach by [13] introduced Fourier-based positional encoding, projecting input coordinates into high-dimensional frequency spaces to mitigate the spectral bias inherent in ReLU-activated networks. This methodology effectively augments the model’s frequency awareness through harmonic decomposition of spatial inputs. Subsequent investigations into activation function design have yielded alternative architectures with improved high-frequency reconstruction capabilities. SIREN [1] and FINER [14] suggested that using periodic functions instead of ReLU activation functions can more effectively capture the fine details of the INR. Remote sensing technology leverages various platforms and sensors to capture images of the Earth’s surface from a distance, utilizing different wavelengths and imaging techniques to meet specific goals [15,16]. This approach distinguishes remote sensing systems from traditional optical systems that rely solely on visible light [17]. Remote sensing platforms, such as satellites and aircraft, operate across diverse frequency bands and modalities, including optical, radar, and thermal sensors, to address a wide range of operational requirements [18,19].

However, optical remote sensing images fundamentally differ from conventional natural images due to their larger spatial extent, denser fine structures, repetitive textures, and more complex scene layouts. These characteristics make accurate representation and reconstruction particularly challenging. Although remote sensing imagery has been widely used in fields such as environmental monitoring, urban planning, and disaster response, the use of Implicit Neural Representations (INRs) for remote sensing images remains underexplored. While INRs perform well on some regions of these images, they often struggle to capture the fine details needed for accurate analysis [1,4], posing challenges in representing complex features such as infrastructure, vegetation, and other fine-grained spatial patterns [20,21].

The ReLU activation function [22], a commonly used nonlinear activation in neural networks, outputs the input when it is greater than or equal to 0 and outputs 0 when it is less than 0, resulting in a dead negative region. These characteristics of one-sided suppression and a wide receptive field maximize neuron selection, enhancing the model’s expressive power and accelerating convergence. Due to the advantages of the ReLU activation function, it is widely applied in various tasks, including object detection, semantic segmentation, image classification, and image super-resolution [23,24]. However, preliminary findings in INR show that the ReLU activation function struggles to represent signals accurately, especially when capturing fine details [23]. We revisit the ReLU activation, widely known for its dead negative region, which can lead to ineffective representations in MLP-based INR models. To address the limitations of ReLU, we propose the Dead-Free Linear Unit (DeLU), which replaces the negative region with the absolute value of the input instead of zero, thereby extending it to a general form with linear transformations and eliminating the ineffective region. Featuring dead-free nonlinearity and allowing for flexible linear adjustments, DeLU enhances the model’s expressive power and signal learning capability for INR. Moreover, SIREN is a seminal work in implicit neural representation, demonstrating that sine functions, which have periodic and oscillatory properties, are particularly well-suited for representing complex signals and their derivatives. Inspired by the success of SIREN in INR, we combine DeLU with SIREN’s periodic activation. To differentiate, we call it DeLU-SIREN in this work. Specifically, we multiply SIREN’s activation function with DeLU, preserving the sine function’s oscillatory behavior along the x-axis while significantly expanding its expressive range. Extensive experiment results on five remote sensing datasets validate the efficacy of our proposed method. In addition to its empirical effectiveness, the proposed DeLU-SIREN addresses a fundamental limitation of existing INR activations for remote sensing imagery. Specifically, it effectively mitigates gradient saturation and provides a stable gradient envelope for modeling high-frequency spatial structures, while extending the dynamic range of periodic representations beyond the bounded amplitude of conventional sine-based activations. This makes the proposed design particularly suitable for remote sensing scenes with sharp structural transitions, radiometric variations, and complex fine-grained patterns.

The main contributions of this work are threefold:

- We identify that dead regions, gradient sparsity, and saturation in conventional activations form a fundamental theoretical bottleneck for modeling high-frequency spatial details in INR-based remote sensing reconstruction.
- We propose DeLU, a mathematically grounded activation function that preserves strictly non-vanishing and constant gradient flow, thereby offering a principled solution to gradient attenuation in fitting rapidly varying signals.
- We further develop DeLU-SIREN, which unifies periodic inductive bias with an unbounded dynamic range, resulting in a more expressive and robust INR framework for remote sensing imagery.

## 2. Related Work

### 2.1. Implicit Neural Representation

Implicit Neural Representations (INRs) employ neural networks to model continuous signals or data, thereby effectively capturing their underlying continuity while enabling compact and efficient representations. Recent advances in INRs have demonstrated considerable potential across a wide range of applications. In computer vision and image processing [13], INRs provide efficient image generation and fine-detail reconstruction capabilities. In audio signal processing [25], they facilitate the modeling and reconstruction of complex acoustic signals. In 3D modeling [3], INRs enable efficient scene representation and realistic rendering. In medical image reconstruction [26], particularly for MRI and CT imaging, they have been used to improve reconstruction quality and accuracy. To further enhance the performance of INRs, a variety of representative methods have been proposed. SIREN [1] employs sinusoidal activation functions to capture high-frequency signals effectively. FINER [14] improves reconstruction resolution through multi-scale frequency decomposition. DINER [27] introduces an architecture designed to adapt to the time-varying characteristics of dynamic signals. H-SIREN [28] enhances high-frequency modeling by incorporating hyperbolic sine functions and demonstrates strong performance in applications such as computer vision and physical simulation. SketchINR [29] utilizes implicit neural models to compress and generate high-fidelity vector sketches. NeSLAM [30] integrates INRs with SDF-based representations for accurate depth estimation and self-supervised tracking. INCODE [31] exploits prior knowledge to regularize the sinusoidal activation functions of INRs, enabling efficient signal representation and denoising. NGEL-SLAM [32] combines implicit fields with octree structures to achieve low-latency and accurate SLAM mapping. Collectively, these studies broaden the applicability of INRs and provide new directions for improving their representation capability across diverse domains. INRs have also attracted increasing attention in the field of remote sensing. HSI-INR [33], which applies INRs to hyperspectral imagery, enables continuous modeling of spectral information and supports efficient spectral reconstruction and analysis while reducing data redundancy, making it particularly valuable for applications such as agriculture, environmental monitoring, and mineral exploration. In 3D remote sensing, INR-based models have been used to reconstruct and represent complex terrains and urban structures. By incorporating implicit geometric representations, these methods substantially improve the compression efficiency and resolution of point cloud data [34]. In addition, INR-based approaches provide a robust framework for multispectral and hyperspectral data fusion, thereby facilitating advances in cross-modal data analysis and synthesis [35].

### 2.2. Remote Sensing Imagery

Remote sensing imagery refers to acquiring information about the Earth’s surface through satellite or airborne sensors, capturing data across various electromagnetic spectra such as visible, infrared, and microwave bands. These sensors, mounted on platforms such as satellites or aircraft, provide high-resolution, large-scale imagery that is invaluable for monitoring land use, environmental changes, and urban development [15,16,36]. Remote sensing imagery enables continuous observation of vast geographic areas, offering detailed insights into both natural landscapes and human activities [18]. Remote sensing systems are deployed on a variety of platforms, from satellites like Sentinel-2 [37], which provides consistent global multispectral coverage, to drones and UAVs, such as in the UAVid dataset [38], which offer high-resolution imagery for localized urban analysis. These platforms allow for systematic data collection across wide regions, enabling researchers to monitor environmental conditions, track urban expansion, and manage natural resources effectively [39]. Numerous datasets have been developed to support advancements in remote sensing research. LandCover.ai [40] provides high-resolution satellite imagery designed explicitly for land cover classification in urban and rural areas. The LoveDA dataset [41] facilitates domain adaptation tasks by providing imagery from diverse environments, bridging the gap between rural and urban settings. ISPRS Potsdam [42] is frequently used in urban planning research, offering detailed aerial imagery for building detection and infrastructure mapping. In environmental monitoring, the BigEarthNet dataset [43] supplies multi-label remote sensing imagery that supports land cover and environmental assessments across multiple European regions. With long-term global observations, the MODIS dataset [44] is a critical resource for tracking vegetation, land cover, and climate-related changes over time. Additionally, the National Agriculture Imagery Program (NAIP) [45] contributes to agricultural monitoring by providing high-resolution imagery for crop and land-use analysis within the United States. In this work, we propose a novel activation function for MLP-based Implicit Neural Representation models applied to remote sensing imagery, aiming to enhance image representation and improve performance on various remote sensing tasks.

## 3. Background and Method

### 3.1. Background

Preliminary of INR. Implicit Neural Representations (INRs) have gained attention as a method for encoding data through continuous functions parameterized by neural networks, contrasting with traditional approaches that rely on explicit geometry. This allows for efficient representation of high-dimensional signals, such as images or 3D shapes, through functions $f:R^{d}\to R^{n}$, where *d* is the input dimension (e.g., spatial coordinates) and *n* is the output dimension (e.g., color or density). To achieve this, a common approach involves using a multi-layer perceptron (MLP) to approximate the underlying signal, represented mathematically as:

(1) $$f\left(x\right)=\sigma (W_{n}\cdot \sigma \left(W_{n-1}\cdot \sigma \left(\ldots \sigma \left(W_{1}\cdot x+b_{1}\right)+b_{n-1}\right)+b_{n}\right)$$

where σ is a non-linear activation function, $W_{i}$ are weight matrices, and $b_{i}$ are bias vectors. The implicit nature of these representations facilitates efficient storage and allows for the continuous evaluation of signals at arbitrary resolutions, making them particularly advantageous for tasks like 3D reconstruction and scene representation.

**Figure 1:**
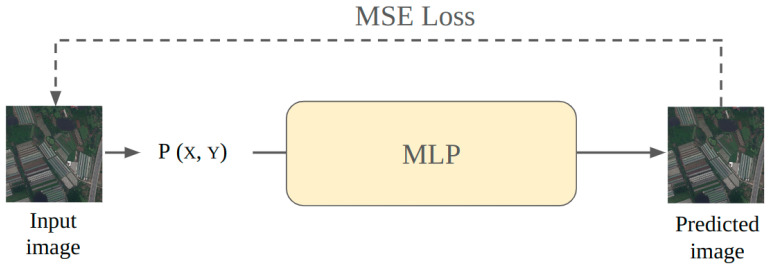
A typical case of using an MLP to learn INRs for images. The pixel coordinates are input into the MLP to produce the corresponding pixel values, and the MSE loss function computes the loss between the ground truth and the predictions to perform backpropagation.

The framework for learning INR for image signals is shown in Figure 1. The network takes pixel coordinates as input and involves minimizing a loss function that quantifies the difference between the predicted outputs and the ground truth data corresponding to the pixel coordinates. This is typically achieved through gradient descent optimization, where the objective is to find the optimal set of parameters θ that minimize the loss L. The optimization can be expressed mathematically as

(2) $$\theta^{*}=\arg\min_{\theta }L\left(f\left(x;\theta \right),y\right)$$

where f(x;θ) represents the network output for input coordinates *x*, and *y* denotes the ground truth values. In the case of image reconstruction, the input coordinates *x* are each pixel’s coordinates relative to the entire image, and the truth values *y* are pixel values (e.g., colors for each RGB channel). By iteratively adjusting the weights and biases based on the gradients of the loss, the network learns to model the underlying signal accurately. The loss function L is typically the Mean Squared Error (MSE), which measures the squared difference between predicted pixel values and truth values. The MSE loss is given by the following

(3) $$\mathrm{MSE}\left(\hat{P},P\right)=\frac{1}{W\cdot H}\sum_{i=1}^{W}\sum_{j=1}^{H}{\left(\hat{P}\left(X,Y\right)-P\left(X,Y\right)\right)}^{2}$$

where *W* and *H* are the image’s width and height, and (X,Y) denote the pixel coordinates. The terms $\hat{P}\left(X,Y\right)$ and P(X,Y) represent the predicted and actual pixel values, respectively.

### 3.2. Dead-Free Linear Unit

**Figure 2:**
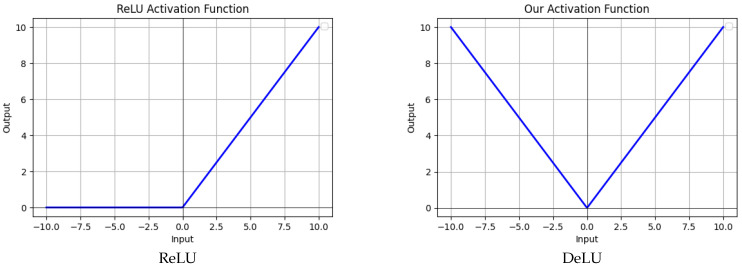
Visualization of the activation functions: ReLU and DeLU.

Activation functions play a pivotal role in neural networks by introducing non-linearity, enabling models to learn and represent complex patterns in data. Among the various activation functions, the Rectified Linear Unit (ReLU) has gained widespread popularity since its introduction, primarily due to its simplicity and effectiveness. These advantages have contributed to its extensive adoption in modern deep-learning frameworks. The mathematical expression for ReLU is:

(4) $$f\left(x\right)=\left\{\begin{array}{cc} x & \mathrm{if}\;x>0\\ 0 & \mathrm{if}\;x\leq 0 \end{array}\right.$$

where *x* represents the input and f(x) denotes the output of the activation function. However, the ReLU function is known to exhibit a dead negative region, becoming non-functional for inputs less than or equal to zero, which results in ineffective representation when learning INRs. To address this limitation, we propose the Dead-Free Linear Unit (DeLU), which extends ReLU using an absolute function and enhances its versatility with linear transformations, thereby eliminating the ineffective region. Our proposed activation function can be formulated as follows,

(5) f(x)=k∥x∥+b

where *k* and *b* are model hyperparameters, and ∥∥ denotes the absolute operation. Our activation function, as shown in Figure 2, introduces non-linearity through the use of an absolute operation, which eliminates dead regions and allows for more efficient and flexible linear adjustments. Additionally, the parameters *k* and *b* enhance the flexibility and adaptability of the function.

### 3.3. DeLU-SIREN

The Sine Representation Network (SIREN) is a seminal INR model that employs periodic sine functions as activation functions, demonstrating remarkable efficacy in learning complex signal approximations. The mathematical representation of a simple SIREN can be articulated as follows,

(6) $$y=W_{n}\cdot \sin\left(W_{n-1}\cdot \sin\left(\ldots \sin\left(W_{1}\cdot x+b_{1}\right)+b_{2}\right)\ldots +b_{n-1}\right)+b_{n},$$

where *x* denotes the input vector, while $W_{i}$ and $b_{i}$ represent the weight matrices and biases, respectively, at each layer *i*. The parameter *n* signifies the total number of layers within the network. The training of SIREN models involves learning parameters through standard backpropagation methods. The use of sine functions introduces periodicity into the network, giving SIREN enhanced representational capability. This makes SIREN particularly valuable in applications such as 3D shape representation, neural rendering, and more.

**Figure 3:**
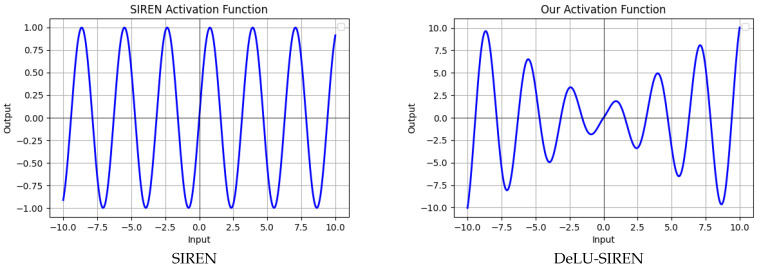
Visualization of the activation functions for SIREN and our proposed DeLU-SIREN.

Based on the success of the periodic activation function SIREN, we combine our activation function with the sine activation function from SIREN to develop a new non-linear activation function. Specifically, we multiply our activation function with the sine activation function to create DeLU-SIREN, which preserves the sine function’s original symmetry and smooth oscillation along the x-axis while gradually increasing its amplitude, as shown in Figure 3. Unlike the sine function employed in SIREN, which has a range of [−1,1] for any real number *x*, our DeLU-SIREN features a range that spans from negative infinity to positive infinity. The DeLU-SIREN can be formulated as follows.

(7) $$\begin{array}{cc} y_{n} & =\beta_{n}\sin\left(\omega_{0}\left(W_{n}x_{n-1}+b_{n}\right)\right),\\ \beta_{n} & =k\left|x_{n-1}\right|+b. \end{array}$$

**Figure 4:**
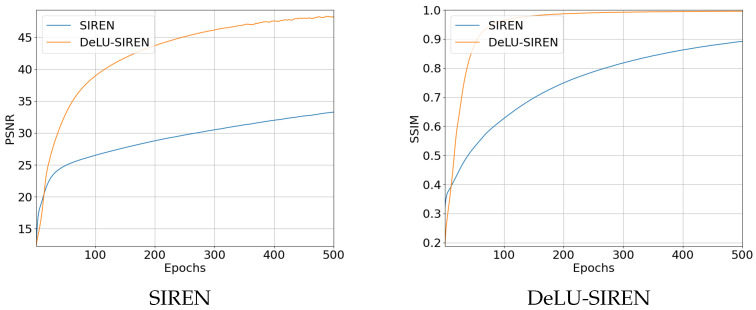
Training curves for PSNR (left) and SSIM (right) of SIREN and DeLU-SIREN models over multiple epochs.

We set SIREN as the baseline and compare its performance with our linear transformations in SIREN for learning INR for images and evaluating reconstruction accuracy using PSNR and SSIM. The results in Table 1 show that DeLU-SIREN improves model performance by 15.01 PSNR and 0.1033 SSIM. Additionally, we plot the training curves of SIREN and DeLU-SIREN over the corresponding epochs, showing PSNR and SSIM accuracy, as shown in Figure 4.

### 3.4. Perspective of Neural Tangent Kernel

The Neural Tangent Kernel (NTK) theory is a theory for understanding the behavior of neural networks and analyzing the training process. Specifically, the Neural Tangent Kernel (NTK) theory treats neural network training as a process similar to kernel-based regression. By studying the network’s NTK, particularly its diagonal characteristics or kernel eigenvalues, one can gain insights into how effectively the network learns and converges to fit the underlying data. In the NTK, a more pronounced diagonal property not only enhances shift-invariance but also contributes to improved convergence rates during training. Moreover, the presence of larger eigenvalues facilitates faster convergence for high-frequency components, thereby optimizing the model’s ability to capture intricate patterns in the data. The NTK is mathematically defined as:

(8) $$\mathrm{NTK}=\nabla_{w}f{\left(x_{i};w\right)}^{T}\nabla_{w}f\left(x_{j};w\right).$$

In this equation, Neural Tangent Kernel (NTK) represents the entry of the NTK matrix corresponding to input samples $x_{i}$ and $x_{j}$, $\nabla_{w}f\left(x_{i};w\right)$ denotes the gradient of the neural network output with respect to the network parameters w for the input $x_{i}$.

**Figure 5:**
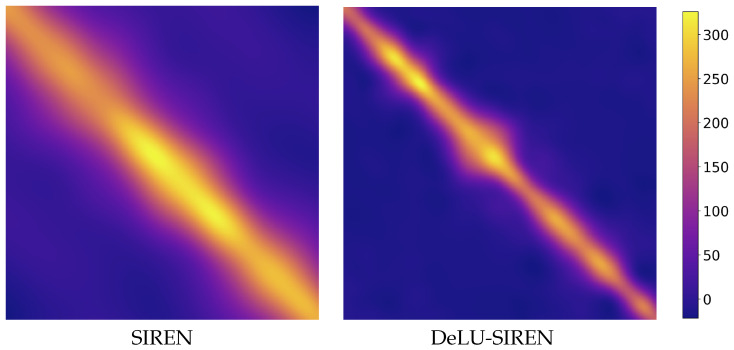
Visualization of the NTK for SIREN and DeLU-SIREN activation function. The strong diagonal characteristics of our method indicate an improved ability to capture high- frequency components.

**Figure 6:**
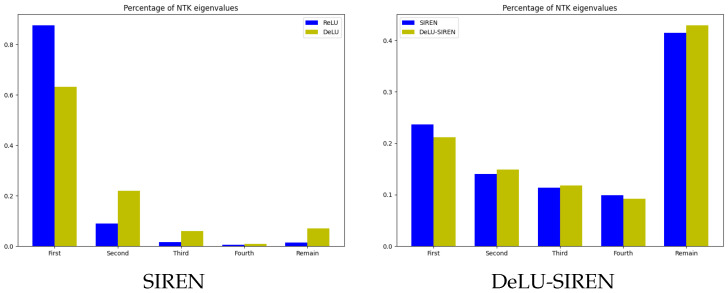
The distribution of NTK eigenvalue magnitudes. First is the percentage of the largest eigenvalue, Second is the percentage of the second-largest, and Remain is the percentage of the rest.

We use a 1D signal with 300 coordinates as the target and visualize the NTK metrics for both SIREN and our DeLU-SIREN. As shown in Figure 5, a comparison of the NTKs reveals a stronger diagonal structure in our DeLU-SIREN, suggesting an enhanced capacity to capture high-frequency details. Additionally, we visualize the percentage contribution of the first four eigenvalues [46], along with the cumulative sum of the remaining eigenvalues, comparing ReLU, SIREN, and their linear transformations: DeLU and DeLU-SIREN. As shown in Figure 6, our linear transformations smooth the distribution of the eigenvalues by reducing the dominance of the first eigenvalue and increasing the others in the empirical NTK matrix, resulting in a more balanced eigenvalue distribution during training.

## 4. Experiments

### 4.1. Implementation Details and Evaluation Metrics

In our experiments, we follow the SIREN model initialization and the Adam optimizer [1]. The experiments were conducted using PyTorch 1.11.0, a widely used deep learning framework. The GPU employed was an NVIDIA RTX A5000, and the learning rate was set to 0.0001. The models were trained for 500 epochs with a batch size of 1.

To evaluate the quality of the restored images, we use two metrics widely used in the field of image restoration: Peak Signal-to-Noise Ratio (PSNR) and Structural Similarity Index (SSIM). These metrics provide quantitative measures to assess the fidelity of restored images compared to original ones. PSNR is a metric that measures the ratio between the maximum possible power of a signal (in this case, the image) and the power of corrupting noise that affects the fidelity of its representation. It is commonly used to evaluate the quality of reconstructed images. PSNR is calculated by dividing the squared maximum possible pixel value by the MSE between the original image and the predicted image. SSIM, on the other hand, is a perception-based model that considers image degradation as a perceived change in structural information. It incorporates luminance, contrast, and structure, providing a more comprehensive view of image quality than PSNR. The local means, variances, and covariances of the original and restored images are utilized to calculate SSIM. Both PSNR and SSIM serve as essential metrics in the realm of image restoration, offering distinct perspectives on image quality. While PSNR provides a straightforward measure of error, SSIM gives insights into perceived structural changes. Employing these metrics together can provide a comprehensive evaluation of image restoration techniques.

### 4.2. Remote Sensing Image Reconstruction

We conduct experiments for image reconstruction on several remote sensing image datasets, including LandCover.ai [40], LoveDA [41], INRIA [47], UAVid [38], and ISPRS Potsdam [48], enabling comprehensive analysis and documentation of the Earth’s surface. Moreover, we randomly select 20 remote sensing images from each dataset as task targets and calculate the average reconstruction accuracy for these images during evaluation. We compare various state-of-the-art methods with our approach to evaluate the effectiveness of our method in remote sensing image reconstruction, including position encoding and ReLU activation function-based INRs (PEMLP) [3], sinusoidal representation networks using sine periodic activation functions (SIREN) [1], learning spatially collaged Fourier bases for implicit neural representation (SCONE) [49], disorder-invariant implicit neural representation (DINER) [27], and flexible spectral bias tuning in implicit neural representations (FINER) [14]. More specifically, in learning INR for remote sensing image reconstruction, the model takes pixel coordinates as input and predicts the corresponding pixel values. We follow the SIREN training hyperparameters and resize the input images to 256 × 256 pixels to train the INR models. Based on our preliminary findings, we set the model activation function parameters *k* and *b* to 3 and 2, respectively, and trained the model for 500 epochs for the remote sensing image reconstruction task.

LoveDA [41] is a dataset specifically designed for urban and rural land cover classification, consisting of 5987 high-quality remote sensing images, each with a resolution of 1024 × 1024 pixels and a spatial resolution of 0.3 m. Each image is meticulously annotated at the pixel level, making it highly suitable for land cover classification tasks. These images encompass seven types of land cover: buildings, roads, water, wasteland, forest, farmland, and ground. The dataset is divided into two subsets based on geographic characteristics: LoveDA-Urban and LoveDA-Rural. LoveDA-Urban focuses primarily on urban scenes, characterized by complex and diverse backgrounds, dense buildings [50], and fewer natural features. In contrast, LoveDA-Rural emphasizes rural areas featuring expansive natural landscapes, sparse man-made structures, and abundant natural features. The significant differences in land cover types, label granularity, and sampling resolution between urban and rural scenes enable researchers to test the performance of deep learning models in different geographical environments, thereby optimizing model robustness and generalizability. With these characteristics, the LoveDA dataset has been widely used in various remote sensing-related fields [51,52], such as urban planning, disaster monitoring, and land use analysis. For example, in urban planning, LoveDA data can be used to identify and track urban expansion and changes in land cover, while in disaster monitoring, precise classification labels help to quickly assess damage in affected areas. The LoveDA dataset also provides a standardized benchmark environment, allowing researchers to easily evaluate and compare the performance of different deep learning models. Evaluation metrics for LoveDA typically include pixel accuracy (PA) [53] and Intersection over Union (IoU) [54], which comprehensively reflect model performance in classification tasks.

**Figure 7:**
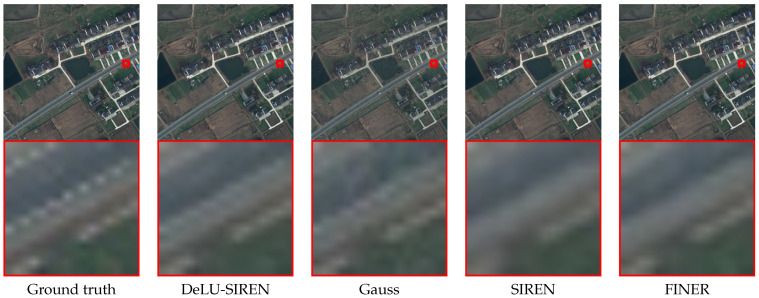
Qualitative comparisons of remote sensing images from the LoveDA dataset between our methods and other approaches.

We present model performance comparisons with advanced methods on the LoveDA dataset test set using remote sensing images, as shown in Table 2. We observe that our DeLU-SIREN achieves state-of-the-art performance with a PSNR of 48.28 and an SSIM of 0.9953, showing an improvement of 15.01 PSNR and 0.0655 SSIM over SIREN. Compared to the latest method, Finer, our DeLU-SIREN also achieves a notable increase of 10.98 PSNR and 0.0418 SSIM in image reconstruction accuracy. Moreover, since the LoveDA dataset includes both urban and rural areas, which have different distributions, we also utilize various INR models to perform reconstruction and compare the results. We observe that our method achieves competitive reconstruction results in both urban and rural scenarios compared to other advanced methods. As shown in Figure 7, our method avoids the noisy artifacts produced by the Gauss model while preserving fine details in the image reconstruction results.

LandCover.ai [40] is a dataset designed for automated land cover classification, primarily applied in semantic segmentation tasks for supervised learning. The dataset consists of aerial imagery from Poland, with all images annotated at the pixel level, covering four main land cover types: buildings, forests, water bodies, and roads. It includes 41 orthophotos, covering approximately 216.27 km^2^, with a resolution of 25 to 50 cm per pixel, ensuring high-precision details. The images are divided into 512 × 512 pixel tiles based on predefined fixed sizes, preserving geographic information to facilitate effective training and validation of deep learning models. Due to the diversity of scenes, which span different geographic regions, climates, and seasons, this dataset is particularly suitable for studying urban-rural land cover changes [55], environmental monitoring [56], and urban planning [57].

**Figure 8:**
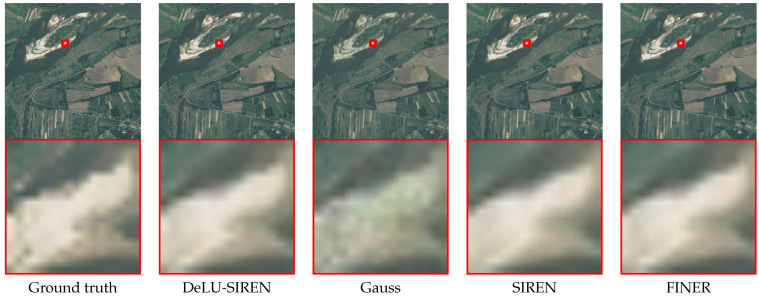
Qualitative comparisons of remote sensing images from the LandCover.ai dataset between our methods and other approaches.

In Table 3, we report performance comparisons with existing methods on the test set of the LandCover.ai dataset with remote sensing images. We observe that our method achieves the best model performance of image reconstruction accuracy with 48.75 PSNR and 0.996 SSIM, which gets 13.45 PSNR and 0.078 SSIM improvement compared with SIREN. Compared to the latest INR model, Finer, our method also achieves an improvement of 10.08 PSNR and 0.040 SSIM. Moreover, we visualize the remote sensing image reconstruction results with details, as shown in Figure 8. We observe that DeLU-SIREN captures more precise image details than other methods, including Finer, particularly in exposed surface areas of remote sensing images.

ISPRS Potsdam is an important dataset designed specifically for urban remote sensing 2D semantic segmentation and object detection tasks. It consists of high-resolution aerial remote sensing images of the Potsdam urban area in Germany. This dataset aims to advance research on detailed classification and segmentation of urban land cover, especially in the field of high-resolution remote sensing image processing. The Potsdam dataset contains 38,782 high-resolution color remote sensing images, each with dimensions of 6000 × 6000 pixels and a spatial resolution of 5 cm per pixel, covering the RGB and near-infrared (NIR) spectral bands. The dataset provides detailed annotations for six land cover classes, including impervious surfaces, buildings, low vegetation, trees, cars, and clutter/background. All images are labeled at the pixel level, providing detailed information on the land cover category of each pixel, which can be used to train and evaluate algorithms for land cover classification, object detection, and semantic segmentation tasks. The main characteristics of the Potsdam dataset are its high resolution and multispectral features, which make it highly accurate for land cover classification and object detection tasks. Moreover, the dataset provides challenging scenarios, such as complex land cover compositions and varying illumination conditions, offering a more realistic testing environment for practical algorithm applications.

**Figure 9:**
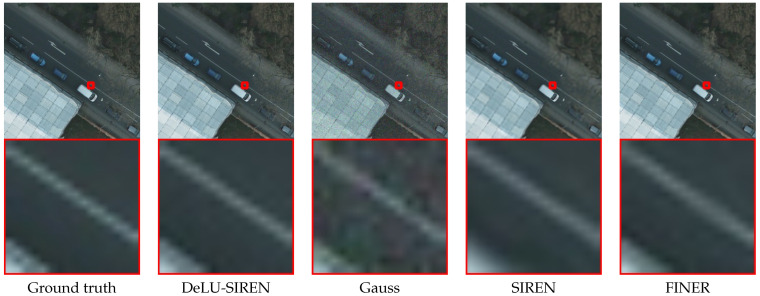
Qualitative comparisons of remote sensing images from the ISPRS Potsdam dataset between DeLU-SIREN and other approaches.

For the ISPRS Potsdam dataset, we evaluate the performance of our method in reconstructing remote sensing images compared to other INR methods. As shown in Table 4, our method achieves a reconstruction accuracy of 49.16 PSNR and 0.9959 SSIM, representing a significant improvement of 13.01 PSNR and 0.074 SSIM over SIREN. Moreover, we visualize the remote sensing image reconstruction results of our method and other methods in detail, as shown in Figure 9. We observe that on the Potsdam remote sensing dataset, our method captures more precise image details than other methods, including the lane markings on roads.

### 4.3. High-Resolution Remote Sensing Image Reconstruction

Remote sensing images often exhibit high resolution, containing a wealth of information within each image, which indicates that the representation functions of these images are more complex. This complexity presents significant challenges for image reconstruction tasks. For the challenging high-resolution image reconstruction task, we evaluate the effectiveness of our method compared to the latest INR methods. In our activation function, we set *k* and *b* to 1 and 5, respectively. For high-resolution image reconstruction, we select images from the UAVid [38] and INRIA datasets, resizing them to 1500 × 900 due to GPU memory limitations. These datasets are designed for urban scene analysis from a drone perspective.

The UAVid [47] dataset enhances the understanding of urban environments in computer vision applications. It consists of high-resolution video frames captured by drones in various urban settings, offering an overhead perspective with resolutions up to 3840 × 2160 pixels. The dataset includes eight semantic classes, such as buildings, roads, trees, and vehicles. UAVid presents challenges due to significant viewpoint variations, scale changes in objects, and complex backgrounds, making it suitable for applications like urban surveillance and autonomous driving assistance systems, which require precise comprehension of urban environments.

**Figure 10:**
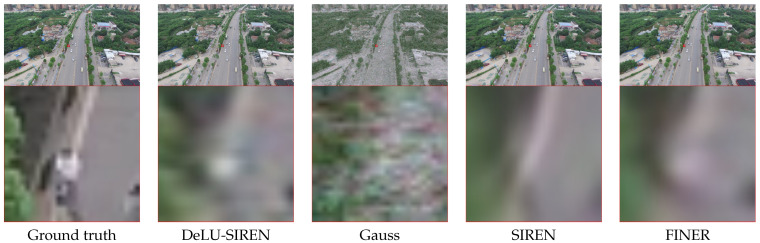
Qualitative comparisons of remote sensing images from the UAVid dataset between our methods and other approaches.

As shown in Table 5, our method performs exceptionally well in high-resolution image reconstruction, achieving 26.88 PSNR and 0.936 SSIM. Compared with SIREN, DeLU-SIREN improves PSNR by 7.58 and SSIM by 0.238, and compared with FINER, it further improves PSNR by 2.13 and SSIM by 0.041, as also evidenced by the qualitative results in Figure 10.

INRIA [47], developed for building segmentation task modules, contains 360 high-resolution RGB images with dimensions of 5000 × 5000 pixels and a spatial resolution of 0.3 m per pixel. Comprising 180 training and 180 testing images, it covers ten cities in the United States and Austria, facilitating the evaluation of model generalization across diverse urban landscapes. Generated through aerial photography, images undergo geometric and radiometric corrections to ensure precise alignment with real-world geographic coordinates. The primary objective is to develop robust models that adapt to varying urban environments, particularly assessing performance under different lighting conditions and architectural styles. The dataset provides binary semantic labels for buildings and non-buildings, establishing a crucial benchmark for building segmentation tasks.

**Figure 11:**
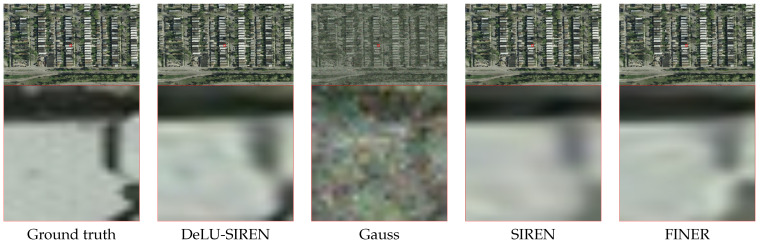
Qualitative comparisons of remote sensing images from the INRIA dataset between our methods and other approaches.

As shown in Table 6, DeLU-SIREN demonstrates outstanding performance in the image reconstruction task on the INRIA dataset, achieving a PSNR of 44.17 and an SSIM of 0.9961. This represents an improvement of 10.32 in PSNR and 0.0309 in SSIM over the SIREN method. Our approach consistently delivers superior image reconstruction quality compared to more recent methods, such as FINER. In the visualized results (Figure 11), our method exhibits enhanced clarity and precision, particularly in reconstructing fine details such as cyclists in urban scenes. The reconstructed images display sharper contours and better-defined structures, ensuring a more accurate representation of moving objects like cyclists, which are often challenging to reconstruct due to their small size and dynamic nature. This improvement highlights our method’s high fidelity in capturing static and dynamic elements across complex urban environments in the INRIA dataset.

### 4.4. Ablation Study of k and b

**Figure 12:**
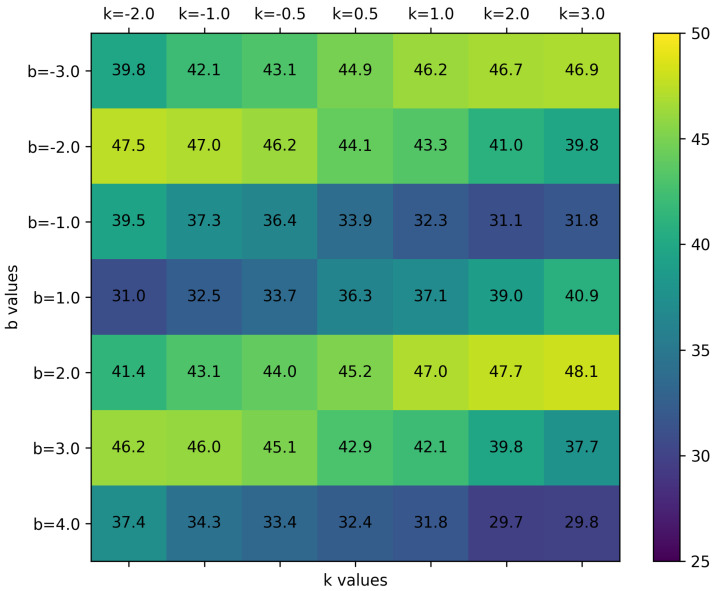
Impact of varying *k* and *b* values on the PSNR of reconstructed images using DeLU-SIREN. A higher brightness indicates greater accuracy of the reconstructed images under the corresponding parameters.

In our DeLU-SIREN, *k* and *b* are important parameters that significantly influence the form of our activation function. We evaluate image reconstruction accuracy across different *k* and *b* values in DeLU-SIREN on an independent validation set to identify the optimal activation function settings for learning implicit neural representations (INRs) for images, as illustrated in Figure 12. To prevent test set leakage, these validation images are strictly excluded from the final test set. Our findings show that both *k* and *b* significantly impact the model’s performance, with the best results being 48.1 PSNR when b=2 and k=3. Additionally, we observed that the model’s performance is not significantly affected by the sign of *k* and *b*; for example, k=−2 and b=−2 also make our activation function effective, obtaining 47.5 PSNR.

However, when the absolute value of *b* is as small as 1 or −1, or as large as 4, the model struggles to accurately learn the INR for images, achieving a maximum accuracy of 40.9 PSNR. Furthermore, higher values of both *k* and *b* degrade the model’s performance. Consequently, we establish b=2 and k=3 as the baseline for reconstructing remote sensing images.

The choice of *k* and *b* reflects a trade-off between signal complexity and optimization stability. In DeLU-SIREN, the modulation amplitude is defined as EQ.(7) indicating that *k* controls the gradient scaling and sensitivity to local variations, while *b* determines the base activation amplitude. For standard-resolution image reconstruction (e.g., 256×256 or 512×512), we recommend k=3 and b=2 as the default setting, since this configuration provides sufficiently strong gradients for fitting high-frequency details while maintaining stable training. For high-resolution reconstruction, we find that a smaller *k* and a larger *b* are more appropriate. This is because high-resolution remote sensing images contain denser and more complex spatial variations, for which a large slope may lead to stronger gradient fluctuations and unstable optimization. Reducing *k* helps regularize the gradient flow, while increasing *b* enlarges the activation amplitude and provides a wider dynamic range for fitting large-scale continuous spatial variations. Accordingly, for high-resolution settings, we adopt k=1 and b=5.

### 4.5. On Dead Region

**Figure 13:**
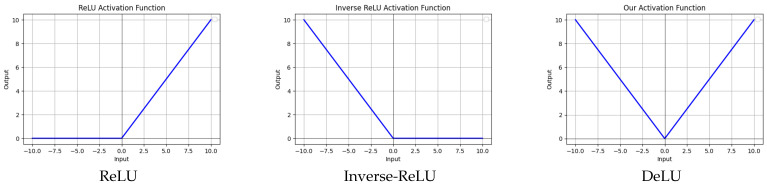
Visualization of activation functions with varying dead regions.

We show that our DeLU can significantly improve the INR performance of seminal work SIREN. Empirically, we find that naively combining ReLU with SIREN yields much worse performance, which is attributed to the dead zone in the original ReLU. Specifically, the ReLU activation function is a non-continuous activation function that exhibits a dead zone for negative input values. To illustrate this, we conduct a study exploring variations in the dead region: one with an activation function where the dead zone is on the left (ReLU), another where the dead zone is on the right (Inverse-ReLU), and a version without a dead region using an absolute value function (DeLU), as shown in Figure 13. From Table 7, we observe that the reconstruction accuracy between Inverse-ReLU and ReLU is similar, while our DeLU yields superior performance.

### 4.6. Uniqueness of DeLU and Compatibility with SIREN

To clarify the theoretical novelty of DeLU and its essential difference from conventional activations such as Tanh, we further analyze its gradient property. In addition, to exclude the possibility that the gain of DeLU-SIREN merely comes from a generic combination of SIREN with arbitrary nonlinearities, we conduct a broader cross-combination study with representative activations.

From a theoretical perspective, the key distinction between DeLU and Tanh lies in gradient preservation for large-magnitude inputs. The derivative of Tanh is given by

(9) $$f^{\prime }\left(x\right)=1-\tanh^{2}\left(x\right),$$

which rapidly approaches zero as |x| increases. In INR-based remote sensing image reconstruction, accurately fitting high-frequency spatial structures often requires large pre-activation responses. Under such conditions, Tanh easily enters the saturation regime, causing severe gradient attenuation and suppressing the learning of fine details. By contrast, DeLU is defined as

(10) f(x)=k|x|+b,

whose derivative is

(11) $$f^{\prime }\left(x\right)=\left\{\begin{array}{cc} k, & x>0,\\ -k, & x<0. \end{array}\right.$$

Therefore, DeLU maintains a constant and non-vanishing gradient magnitude over the entire input domain, avoiding the damping effect of smooth saturating activations.When combined with SIREN, although the periodic nature of the sine component inherently causes the derivative to oscillate, the linear scaling of DeLU preserves a more stable overall gradient envelope, preventing severe gradient attenuation.This property makes it particularly suitable for INR tasks that rely on stable gradient flow to recover high-frequency structures.

To support the above analysis, we further compare DeLU with several standard activations on the basic INR reconstruction task. The results are summarized in Table 8. Although Tanh does not suffer from a hard dead region, its gradient saturation leads to the weakest reconstruction performance, indicating that avoiding hard truncation alone is insufficient. In contrast, DeLU achieves the best performance, demonstrating that its advantage is not simply due to symmetry or smoothness, but to its ability to preserve effective gradient flow for high-frequency fitting.

Furthermore, we investigate whether other activations can also enhance SIREN in a similar manner. Specifically, besides the original SIREN baseline, we construct ReLU-SIREN, Leaky ReLU-SIREN, and Tanh-SIREN under the same network architecture and training protocol. The quantitative results are reported in Table 9. It can be observed that simply combining SIREN with conventional activations does not consistently improve performance. In fact, ReLU-SIREN and Leaky ReLU-SIREN both degrade the original SIREN, while Tanh-SIREN provides only a marginal improvement. In sharp contrast, DeLU-SIREN yields a decisive gain.

This result suggests that the effectiveness of DeLU-SIREN is not due to arbitrary activation stacking, but to a specific structural compatibility. ReLU-style truncation destroys the continuity of sinusoidal oscillations, while Leaky ReLU still introduces asymmetric piecewise behavior that is not well aligned with periodic representations. Although Tanh is smooth, its bounded output and saturating gradient restrict the dynamic range and optimization efficiency of SIREN. In contrast, DeLU preserves symmetry through the absolute value form, while the linear parameters *k* and *b* provide a continuously adjustable amplitude modulation. As a result, DeLU-SIREN simultaneously preserves the periodic inductive bias of SIREN and enhances its dynamic range and gradient stability. These results indicate that DeLU is fundamentally different from conventional activations such as Tanh, and that its effectiveness in INR arises from stable gradient preservation. They further show that the success of DeLU-SIREN is due to principled functional compatibility rather than arbitrary activation stacking.

### 4.7. Uniqueness of DeLU

We compare DeLU with classical ReLU and its variant Leaky ReLU to highlight its unique characteristics. Unlike ReLU and its variants, which utilize a dead region to produce nonlinear features, DeLU incorporates an absolute value operation to provide nonlinearity, along with the hyperparameters *k* (slope) and *b* (bias), as shown below:

(12) f(x)=k∥x∥+b

The absolute value ensures that the function avoids dead zones, while *k* and *b* enable DeLU to possess a broader representational range and controllable first-order gradients, thus effectively representing the rich and complex remote sensing image. Moreover, we provide a comparison with other activation functions in terms of computational complexity, ease of implementation, and impact on the training process. Since our method is a linear transformation of ReLU, its complexity remains O(N), the same as that of ReLU. In terms of ease of implementation, we compare DeLU with other activation functions regarding the computation speed, as shown in Table 10. As reported, DeLU records an inference time of 119 μs, incurring a marginal computational overhead of roughly 12% compared to standard ReLU (106 μs). Although the absolute value and linear scaling operations introduce this slight delay, the substantial gains in reconstruction accuracy make this modest overhead a highly worthwhile trade-off throughout the process of learning INRs for images. In the end, we mathematically demonstrate that our method is more suitable for representing remote sensing images compared to ReLU. The ReLU activation function is defined as

(13) ReLU(x)=max(0,x),

with its derivative given by

(14) $$\frac{\partial }{\partial x}\mathrm{ReLU}\left(x\right)=\left\{\begin{array}{cc} 1, & x>0,\\ 0, & x\leq 0. \end{array}\right.$$

When x≤0, the gradient becomes zero, leading to the well-known “dead neuron problem,” where certain neurons stop contributing to the learning process. This issue significantly hinders the training of implicit neural representations, particularly for learning high-frequency components of signals. In contrast, the activation function DeLU is defined as

(15) DeLU(x)=k|x|+b,

with its derivative expressed as

(16) $$\frac{\partial }{\partial x}\mathrm{DeLU}\left(x\right)=\left\{\begin{array}{cc} k, & x>0,\\ -k, & x<0. \end{array}\right.$$

This ensures that the gradient remains non-zero across the entire domain, except at x=0, where it is not differentiable. The key advantage of this property lies in its ability to avoid vanishing gradients, as the gradient’s magnitude remains constant (*k*) regardless of the input value. This stability in gradient flow facilitates efficient learning, particularly for capturing high-frequency components in complex signals. Implicit neural representations require the network to model both low-frequency and high-frequency features of input data. While ReLU’s sparsity in gradients limits its ability to propagate high-frequency information effectively, DeLU maintains robust gradient flow, preserving high-frequency spectral features. Moreover, the constant and symmetric nature of the gradient ensures stable parameter updates, making DeLU particularly advantageous for tasks involving intricate signal representations such as remote sensing images.

### 4.8. DeLU vs. Variants of ReLU

**Figure 14:**
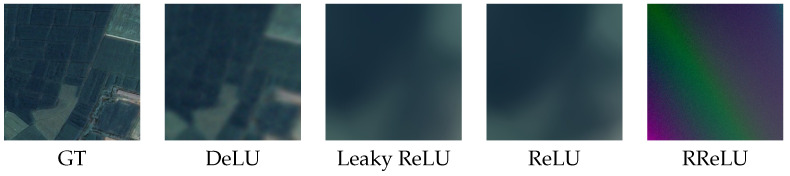
Qualitative comparison of image representation with variants of the ReLU activation function.

We compare DeLU with other variants of the ReLU function in representing remote sensing images. The ReLU variants include Leaky ReLU [58] and RReLU [59], classical activation functions designed to avoid the dead region when the input is less than zero, and their effectiveness has been widely validated across various tasks [59]. We also apply absolute processing, similar to that in our DeLU, to these activation functions to explore the impact of the absolute value operation. As shown in Table 11, DeLU outperforms the other variants of the ReLU function, including their absolute forms. Moreover, to clearly demonstrate the competitiveness of DeLU in representing remote sensing images, we visualize the representation results learned using DeLU and other activation functions, as shown in Figure 14. We observe that, although variants of the ReLU activation function are not well-suited for representing image signals, DeLU significantly outperforms other activation functions.

### 4.9. Performance Gain Without Parameter Increase

As illustrated in numerous studies, increasing the number of neurons in a network’s hidden layers can improve model performance. However, such capacity increases often come at the cost of higher memory consumption and greater computational complexity. These drawbacks are particularly concerning for real-world applications on resource-constrained devices or large-scale deployments. In contrast, our approach modifies without increasing the number of trainable parameters, achieving significant performance improvements. This strategy is particularly valuable when considering resource efficiency, as it enables us to enhance performance while maintaining a compact model architecture. An illustrative comparison of the effects of increasing the network size versus adopting our DeLU activation is provided in Table 12. We compare standard ReLU networks at three different scales (256, 512, and 1024 neurons in each hidden layer) with our DeLU using only 256 neurons. Despite having fewer parameters than the larger ReLU networks, our DeLU-based model achieves superior PSNR and SSIM.This remarkable result highlights the effectiveness of our activation function in capturing fine details and preserving structure without inflating the model’s size.

### 4.10. Ablation Study on Scaling Parameters

To isolate the specific contribution of the dead-free nonlinearity from the added scaling flexibility, we evaluated a Scaled ReLU baseline formulated as f(x)=k·max(0,x)+b. This baseline shares the exact degrees of freedom and initialization as our proposed DeLU.

As shown in Table 13, the addition of scaling parameters only marginally improves standard ReLU in the MLP architecture, still falling significantly short of DeLU. More importantly, when integrated into the SIREN architecture, the Scaled ReLU-SIREN suffers a catastrophic optimization collapse (PSNR dropping to 12.60 dB). This failure occurs because the asymmetric nature of standard ReLU introduces severe variance shifts across hidden layers, destroying the delicate distribution balance required by periodic representations. Conversely, DeLU leverages the absolute value operation to preserve structural symmetry, enabling stable optimization while fully harnessing the representational power of the scaling parameters. This confirms that the primary performance leap is fundamentally driven by the absolute value operation.

## 5. Conclusions

In this work, we revisit the ReLU activation function and address the widely known issue of its dead negative region by introducing a simple yet effective absolute function. Our experiments show that this approach enhances the signal representation capability of ReLU-based models for remote sensing images. Furthermore, our findings indicate that integrating our activation function with state-of-the-art periodic activations can yield even better results. Extensive experiments on five diverse remote sensing datasets consistently verify the effectiveness of DeLU-SIREN. Compared with the baseline SIREN, the proposed method achieves approximately 10-15 dB PSNR improvement, highlighting the importance of stable gradient preservation for high-frequency remote sensing image representation. Despite these promising results, the current framework still depends on manual hyperparameter tuning of *k* and *b*, and its pure MLP-based formulation may limit scalability in cross-modal or large-scale remote sensing applications. Future research will investigate adaptive parameterization and hybrid multi-scale representations to further enhance flexibility, scalability, and generalization.

## Figures and Tables

**Table 1:** Experimental comparison on image representation performance of SIREN and DeLU-SIREN.

Methods	PSNR	SSIM
SIREN [1]	33.27	0.8920
DeLU-SIREN	48.28	0.9953

**Table 2:** Experimental comparison of image reconstruction accuracy for various INR models on the LoveDA dataset. The best results are indicated in bold.

Dataset	Metrics	PEMLP [3]	SIREN [1]	FINER [14]	DINER [27]	SCONE [49]	DeLU-SIREN
LoveDA	PSNR	24.49	33.27	37.30	39.72	40.66	48.28
	SSIM	0.5154	0.9287	0.9532	0.9866	0.9825	0.995
LoveDA-Urban	PSNR	24.53	33.13	37.28	38.92	40.23	48.10
	SSIM	0.5356	0.9134	0.9501	0.9818	0.9778	0.995
LoveDA-Rural	PSNR	24.39	33.48	37.36	41.79	41.22	48.79
	SSIM	0.4971	0.8963	0.9525	0.9859	0.9953	0.9967

**Table 3:** Quantitative results on the LandCover.ai dataset.

Dataset	Metrics	PEMLP [3]	SIREN [1]	FINER [14]	DINER [27]	SCONE [49]	DeLU-SIREN
LandCover.ai	PSNR	25.99	35.30	38.67	41.90	44.12	48.75
	SSIM	0.5751	0.9186	0.9913	0.9561	0.9903	0.9969

**Table 4:** Experimental comparison of image reconstruction accuracy for various INR models on the ISPRS Potsdam dataset. The best results are indicated in bold.

Dataset	Metrics	PEMLP [3]	SIREN [1]	FINER [14]	DINER [27]	SCONE [49]	DeLU-SIREN
ISPRS Potsdam	PSNR	26.70	36.15	39.59	43.19	46.68	49.16
	SSIM	0.6312	0.9213	0.9625	0.9841	0.9930	0.9959

**Table 5:** Experimental comparison of high-resolution image reconstruction accuracy for various INR models on the UAVid dataset. The best results are indicated in bold.

Dataset	Metrics	PEMLP [3]	SIREN [1]	FINER [14]	DeLU-SIREN
UAVid	PSNR	20.71	24.75	25.66	26.88
	SSIM	0.749	0.895	0.915	0.936

**Table 6:** Experimental comparison of high-resolution image reconstruction accuracy for various INR models on the INRIA dataset. The best results are indicated in bold.

Dataset	Metrics	PEMLP [3]	SIREN [1]	FINER [14]	DINER [27]	SCONE [49]	DeLU-SIREN
INRIA	PSNR	21.98	33.85	39.38	38.94	41.16	44.17
	SSIM	0.5259	0.9652	0.9893	0.9929	0.9935	0.9961

**Table 7:** Effect of dead regions.

Methods	PSNR	SSIM
ReLU	22.12	0.4275
Inverse-ReLU	21.82	0.4159
DeLU	24.60	0.5132

**Table 8:** Comparison of DeLU with conventional activations on the basic INR reconstruction task.

Methods	PSNR (dB)	SSIM
Tanh	19.45	0.3612
Leaky ReLU	21.71	0.4143
ReLU	22.12	0.4275
DeLU (Ours)	24.60	0.5132

**Table 9:** Extensive comparison of different activation combinations with SIREN on remote sensing image reconstruction.

Combined Methods	PSNR (dB)	SSIM
ReLU-SIREN	28.15	0.7512
Leaky ReLU-SIREN	31.42	0.8245
SIREN	33.27	0.8920
Tanh-SIREN	34.10	0.9015
DeLU-SIREN (Ours)	48.28	0.9953

**Table 10:** Comparison of inference time and PSNR across variants of ReLU activation functions.

Metrics	ReLU	Leaky ReLU	DeLU
PSNR (db)	22.12	21.71	24.6
Inference time (µs)	106	98	119

**Table 11:** Experimental comparison of variants of the ReLU function. The “Abs” refers to the absolute value operation.

Methods	PSNR	SSIM
RReLU	13.92	0.2438
Abs-RReLU	19.89	0.3642
Leaky ReLU	21.71	0.4143
Abs-Leaky ReLU	22.49	0.4265
ReLU	22.12	0.4275
DeLU (Ours)	24.60	0.5132

**Table 12:** Influence of increasing the number of network parameters in learning INR.

Methods	Number of Neurons	Parameters	PSNR	SSIM
ReLU	256	0.199 M	22.12	0.4275
ReLU	512	0.791 M	22.93	0.4392
ReLU	1024	3.15 M	23.77	0.4512
DeLU (Ours)	256	0.199 M	24.60	0.5132

**Table 13:** Performance comparison controlling for scaling parameters (k=3,b=2).

Architecture	Activation Function	PSNR	SSIM
Standard MLP	Scaled ReLU	22.74	0.4364
Standard MLP	DeLU (Ours)	24.60	0.5132
SIREN	Scaled ReLU-SIREN	12.60	0.3254
SIREN	DeLU-SIREN (Ours)	48.28	0.9953

## Data Availability

The LoveDA dataset utilized in this study is openly accessible at https://github.com/junjue-wang/loveda, accessed on 13 October 2024; the UAVid dataset can be found at https://uavid.nl, accessed on 13 October 2024; the LandCover.ai dataset is available for free at https://landcover.ai.linuxpolska.com/download/landcover.ai.v1.zip, accessed on 13 October 2024; the Potsdam dataset is available at https://drive.google.com/file/d/1uw9nkk5H3dBvirkk0Wau0wjFxQu0xnzR/view?usp=sharing, accessed on 15 October 2024; and the INRIA dataset can be accessed at https://project.inria.fr/aerialimagelabeling/, accessed on 15 October 2024. The core implementation scripts are available from the corresponding author upon reasonable request.

## Abbreviations

The following abbreviations are used in this manuscript:

INRs	Implict neural representations
NRL	Noise-first residual learning
MLP	Multi-layer perceptron
MSE	Mean squared error
SIREN	Sinusoidal Representation Network
PEMLP	Position embedding multi-layer perceptron
FINER	Flexible spectral bias tuning
WIRE	Wavelet implicit neural representations
PSNR	Peak signal-to-noise ratio
SSIM	Structural similarity index
MSFA	Multi-Stage Filter Augmentation
CNN	Convolutional neural network
ReLU	Rectified linear unit
HVS	Human visual system
